# 13-Acetoxysarcocrassolide Induces Apoptosis on Human Gastric Carcinoma Cells Through Mitochondria-Related Apoptotic Pathways: p38/JNK Activation and PI3K/AKT Suppression

**DOI:** 10.3390/md12105295

**Published:** 2014-10-23

**Authors:** Ching-Chyuan Su, Jeff Yi-Fu Chen, Zhong-Hao Din, Jui-Hsin Su, Zih-Yan Yang, Yi-Jen Chen, Robert Y.L. Wang, Yu-Jen Wu

**Affiliations:** 1 Antai Medical Care Cooperation Antai Tian-Sheng Memorial Hospital, Pingtung 92842, Taiwan; E-Mail: a081001@mail.tsmh.org.tw; 2 Department of Beauty Science, Meiho University, Pingtung 91202, Taiwan; 3 Department of Biotechnology, Kaohsiung Medical University, Kaohsiung, Taiwan; E-Mail: yifuc@kmu.edu.tw; 4 Graduate Institute of Applied Healthy and Biotechnology, Meiho University, Pingtung 91202, Taiwan; E-Mail: nmm10023@yahoo.com.tw; 5 National Museum of Marine Biology and Aquarium, Pingtung 94446, Taiwan; E-Mail: x2219@nmmba.gov.tw; 6 Graduate Institute of Food Science, National Pingtung University of Science and Technology, Pingtung 91202, Taiwan; E-Mail: vian10045@gmail.com; 7 Department of Physical Medicine and Rehabilitation, Kaohsiung Medical University Hospital, Kaohsiung, Taiwan; E-Mail: chernkmu@gmail.com; 8 Department of Biomedical Sciences and Research Center for Emerging Viral Infections, College of Medicine, Chang Gung University, Taoyuan 33302, Taiwan

**Keywords:** 13-acetoxysarcocrassolide, soft coral, gastric cancer cells, apoptosis, p38 and JNK pathways

## Abstract

13-acetoxysarcocrassolide (13-AC), an active compound isolated from cultured Formosa soft coral *Sarcophyton crassocaule*, was found to possess anti-proliferative and apoptosis-inducing activities against AGS (human gastric adenocarcinoma cells) gastric carcinoma cells. The anti-tumor effects of 13-AC were determined by MTT assay, colony formation assessment, cell wound-healing assay, TUNEL/4,6-Diamidino-2-phenylindole (DAPI) staining, Annexin V-fluorescein isothiocyanate/propidium iodide (PI) staining and flow cytometry. 13-AC inhibited the growth and migration of gastric carcinoma cells in a dose-dependent manner and induced both early and late apoptosis as assessed by flow cytometer analysis. 13-AC-induced apoptosis was confirmed through observation of a change in ΔΨm, up-regulated expression levels of Bax and Bad proteins, down-regulated expression levels of Bcl-2, Bcl-xl and Mcl-1 proteins, and the activation of caspase-3, caspase-9, p38 and JNK. Furthermore, inhibition of p38 and JNK activity by pretreatment with SB03580 (a p38-specific inhibitor) and SP600125 (a JNK-specific inhibitor) led to rescue of the cell cytotoxicity of 13-AC-treated AGS cells, indicating that the p38 and the JNK pathways are also involved in the 13-AC-induced cell apoptosis. Together, these results suggest that 13-AC induces cell apoptosis against gastric cancer cells through triggering of the mitochondrial-dependent apoptotic pathway as well as activation of the p38 and JNK pathways.

## 1. Introduction

Gastric cancer (GC), a gastrointestinal cancer, is ranked as the second most common cause of cancer-related death in the world [1,2]. It has been reported that estimates of nearly one hundred thousand new cases of gastric cancer have occurred each year since 2010 [3]. The incidence of gastric cancer in East Asia is much higher than that in other regions [4]. Many studies have revealed that the high mortality rate of gastric cancer is related to the lack of an effective therapy for advanced stages of the disease. Many conventional therapy options have been developed for the treatment of gastric cancer, including surgery, chemo- and radiation therapy and combination treatments. The most effective medical treatment is surgical removal of the tumor in the early stages [5]. However, because of occult symptoms in the early stages of gastric cancer, most patients are not diagnosed until the cancer has progressed to an advanced stage. In addition, tumors will recur in many patients receiving surgical resection, leading to short survival durations. The 5-year survival rate has remained at around 20%–25% in the Western world [6,7]. The high mortality rate emphasizes the need for effective medical treatments for patients with advanced stages of gastric cancer [8]. The risk factors for the development of GC are atrophic gastritis, helicobacter pylori infection, intestinal metaplasia, smoking and genetic differences [9]. Although several new drugs, including taxane paclitaxel, platinum derivative oxaliplatin, and the topoiosmerase-I inhibitor irinotecan, have led to a better prognosis for patients with advanced gastric cancer, the response rate is only 20%–40% [10]. Therefore, it is essential to find new treatments for gastric cancer.

Several new therapeutic applications of natural products obtained from soft coral have been widely investigated for the treatment of different types of cancer [11,12,13]. Some chemical compounds isolated from marine soft corals, such as diterphenoids, diterpenes, and prostanoids, have been reported to exert various biological activities, including anti-proliferation, anti-migratory, and apoptosis induction effects, on different cancer cell lines, such as prostate cancer cells, hepatocellular carcinoma, breast cancer cells, colon cancer cells, cervix cancer cells, oral squamous cell, bladder cancer cells and melanoma cells [14,15,16,17,18,19,20]. Soft corals have therefore emerged as one of the most prolific sources of natural products with novel activities and antitumor effects.

When cells are attacked by pathogens, cellular apoptosis can serve as a guard against pathogens and regulate the cell death process during tissue development, in addition to maintaining homeostasis [19,21]. Cells trigger the apoptosis event through the plasma membrane (extrinsic pathways) and/or within cells (intrinsic pathways) [22]. The initiation of intrinsic pathways is caused by the stimulation of biochemical events, resulting in the organelles inside the cells experiencing enhanced intracellular stresses. In this pathway, mitochondria and the endoplasmic reticulum (ER) play important roles in the execution of apoptosis [23,24]. Mitochondria are organelles that participate in energy conversion and play important roles in many cellular functions, including calcium buffering, regulation of signal cascades, and apoptosis regulation. Dysfunction of mitochondria has been found to be the major event during cellular apoptosis. Bcl-2 protein, the pro-apoptotic gene, participates in forming pores and causing a permeable membrane, leading to the release of cytochrome C protein [25]. The cytochrome C protein then activates caspase-9 as well as the downstream effector caspase-3, subsequently cleaving poly (ADP-ribose) polymerase-1 (PARP-1), inducing chromatin condensation and DNA chromatin fragmentation [26]. ER is another important cellular homeostasis principal site, as it regulates protein synthesis, protein folding and intracellular calcium levels [27]. The unfolded protein response (UPR) is a cellular stress response related to ER stress. If the ER stress becomes prolonged and severe, it will ultimately lead to apoptosis [28,29]. Three major transducers mediate this response: PKR-like ER-associated kinase (PERK), activating transcription factor 6 (ATF6), and inositol-requiring enzyme-1 (IRE1) [30,31].

It is important to explore new effective anticancer drugs and develop therapies against gastric cancer. In this study, 13-acetoxysarcocrassolide (13-AC) was isolated from cultured Formosa *Sarcophyton crassocaule* and the cytotoxic effect on gastric carcinoma AGS cells was subsequently examined. 13-AC possessed anti-proliferative, anti-migratory and apoptosis-inducing activities against AGS cells. The results showed that apoptosis was induced by 13-AC through the mitochondrial-dependent apoptotic pathway with p38 and JNK activation. These results provide useful information regarding the biochemical aspects of the cytotoxic effects of 13-AC on AGS cells and will accelerate drug development and improve the monitoring of human gastric carcinoma.

## 2. Results

### 2.1. The Cytotoxic Effects of 13-AC on AGS Gastric Carcinoma Cells

**Figure 1:**
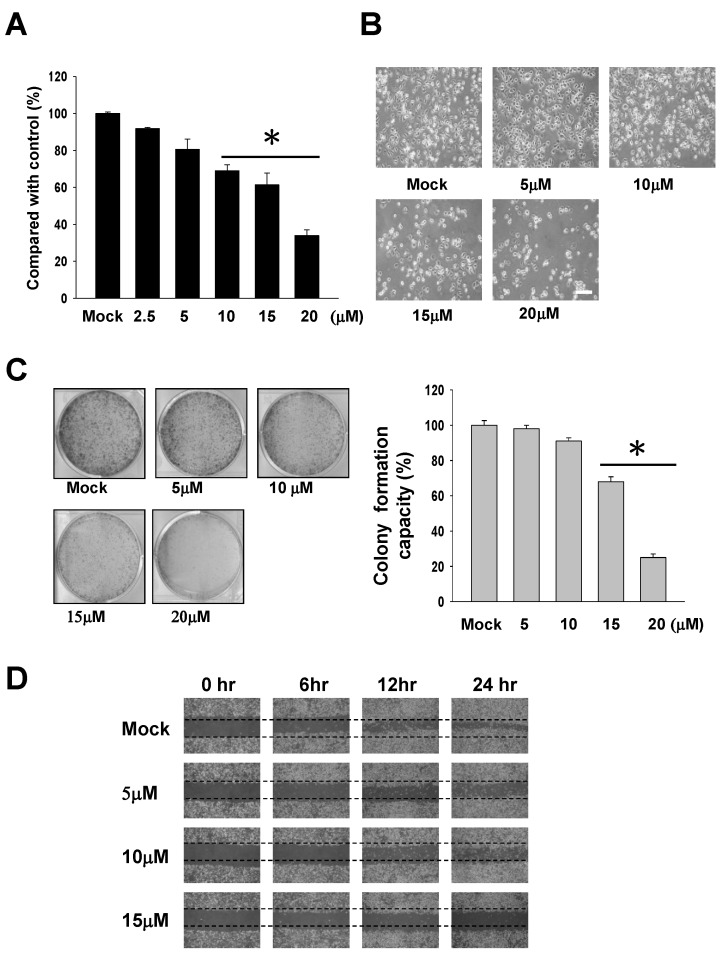
Evaluation of the anti-proliferative and anti-migratory effects of 13-AC on AGS cells (human gastric adenocarcinoma cells). (**A**) The AGS cell viability was suppressed in a dose-dependent manner upon treatment with 13-AC. AGS cells were treated without or with 13-AC at final concentrations between 2.5 μM and 20 μM for 24 h. The cells were then harvested for MTT assay as described in the Materials and Methods section. The data shown are representative of three independent experimental results (* *p* < 0.001). (**B**) The morphological changes of AGS cells upon 13-AC treatment. AGS cells were treated with 5, 10, 15 and 20 μM of 13-AC for 24 h. The morphology of the cells was observed using inverted light microscopy. Scale bars = 20 μm. (**C**) Colony formation assay for AGS cells. AGS cells were treated with various concentrations of 13-AC (5, 10 and 20 μM), followed by a colony formation experiment, as described in the Materials and Methods section. The decreased number of colonies indicated dose-dependent inhibition of AGS cells colony formation upon treatment with 13-AC (* *p* < 0.001). (**D**) Inhibition of AGS cell migration upon treatment with 13-AC. AGS cells were treated with DMSO (control) or 13-AC at a final concentration of 15 μM, followed by examination of cell migration using the cell migration assay as described in the Materials and Methods section. The image was obtained under 100× magnification.

To explore the potential cytotoxic effect of 13-AC on AGS cells, MTT assays, cell morphology assessments, colony formation assays and wound-healing assays were performed. As expected, 13-AC treatment clearly reduced the cell viability of AGS cells in a dose-dependent manner in comparison with the mock control (Figure 1A). Note that a significant reduction (greater than 36%) in cell viability was observed in the 13-AC-treated cells at the final concentration of 20 μM. Next, the cell morphology was investigated using inverted light microscopy. As shown in Figure 1B, the 13-AC-treated AGS cells reduced in size, and a distinct decrease in the cell population was observed in comparison with the mock-treated cells, revealing that 13-AC induces cell apoptosis (Figure 1B). We then tested the colony-forming ability of the 13-AC-treated AGS cells. The results showed a significant decrease in colony formation upon 13-AC treatment. Treatment with 5, 10, 15 and 20 μM of 13-AC dose-dependently reduced colony formation, the reduction rates being approximately 5%, 10%, 31% and 78%, respectively (Figure 1C), indicating the effect of 13-AC on the reduction of colony formation. As the behavior of cancer cell migration is one of the critical processes in the development of programmed cell death, we then evaluated the effect of 13-AC on AGS cell migration using wound-healing assays. The results of the wound-healing migration assays showed that 13-AC treatment led to reduced wound closure in a dose-dependent manner at the 24-hour time point (Figure 1D). 

Similarly, two additional gastric cancer cell lines (NCI-N87 and SNU-1) were chosen for treatment with 13-AC at final concentrations of 5, 10 and 15 μM. The MTT assay results showed that 13-AC treatment of NCIN87 and SNU-1 cells also induced cell cytotoxicity (Supplementary Figure S1). Together, these results implied cell cytotoxic effects of 13-AC on these gastric cancer cells.

### 2.2. 13-AC Induces Apoptosis of AGS Cells

**Figure 2:**
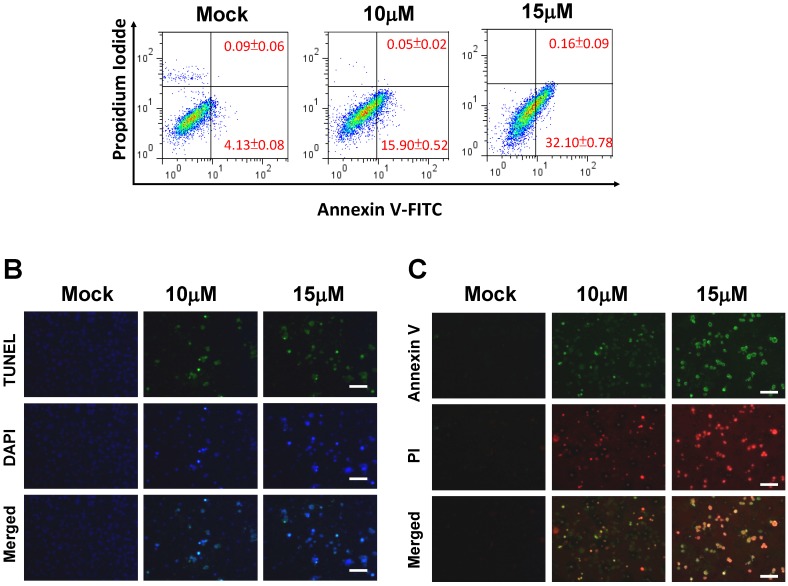
The appearance of apoptosis characteristics in 13-AC-treated AGS cells. (**A**) Detection of apoptotic AGS cells after 10 and 15 μM 13-AC treatment using Annexin V-FITC/PI analysis. Note that early apoptotic cells were increased after 10 and 15 μM 13-AC treatment. (**B**) Detection of apoptotic AGS cells by TUNEL and DAPI staining assay. AGS cells were treated with DMSO or 13-AC at final concentrations of 10 and 15 μM for 24 h and then observed using a fluorescent microscope. Scale bar = 50 μm. (**C**) Annexin V-FITC and PI analyses of apoptotic AGS cells upon 13-AC treatment. AGS cells were stained with Annexin-V (green) as well as PI (red) after 10 and 15 μM 13-AC treatment. Scale bar = 50 μm.

In our previous study, 13-AC induced apoptosis in bladder cancer BFTC cells [20]. To investigate whether 13-AC induces apoptosis of AGS cells, an apoptotic assay was employed. First, AGS cells were stained with fluorescein isothiocyanate (FITCH-labelled Annexin V, green fluorescence) and simultaneously with dye exclusion of propidium iodide (PI) for apoptosis flow cytometric detection in early apoptotic cells analyses. The dose-dependent apoptosis rates were 4.13%, 15.9% and 32.1% when treated with 13-AC at concentrations of 0, 10 and 15 μM, respectively (Figure 2A). These results clearly indicated that 13-AC efficiently induced early apoptosis of AGS cells. Second, TUNEL/DAPI staining and Annexin V-FITC/PI double staining were employed to further validate the apoptotic effect of 13-AC on AGS cells. Some massive apoptotic bodies were observed in AGS cells treated with 10 μM and 15 μM of 13-AC (Figure 2B,C). In contrast, there was neither positive staining with TUNEL/DAPI nor Annexin V-FITC/PI staining in the mock-treated AGS cells. Together, these results demonstrated that treatment with 13-AC significantly induced early apoptosis of AGS cells, and this apoptotic effect was exerted in a dose-dependent manner.

### 2.3. 13-AC Induces Apoptosis and Causes a Mitochondria Membrane Potential Change in AGS Cells

**Figure 3:**
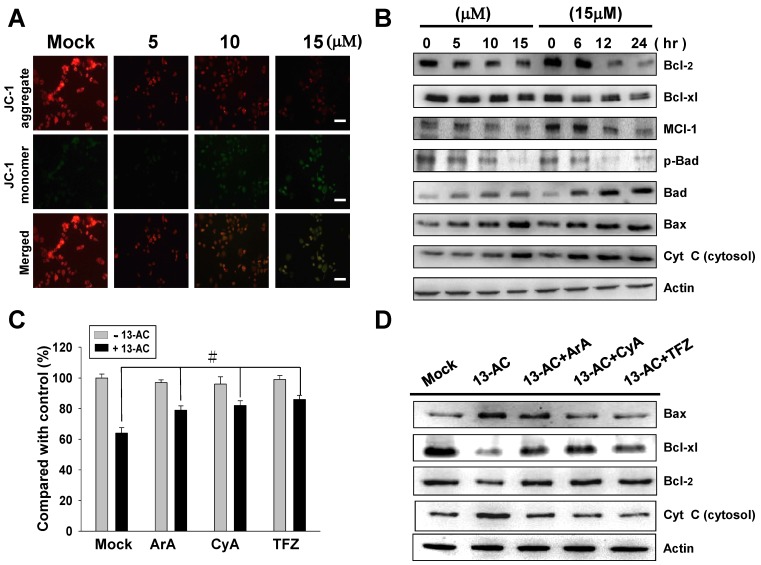
13-AC induces mitochondrial membrane depolarization in AGS cells. (**A**) Cells were treated as indicated and then stained with JC-1 dye, followed by incubation for 20 min at 37 °C, 5% CO_2_. The cells were then imaged under a fluorescence microscope at emission wavelengths of 580 nm (red, upper panels) and 530 nm (green, lower panels). Scale bars = 50 μm. (**B**) Differential expressions of proteins involved in the mitochondrial function were detected by western blotting. Changes in Bcl-2, Bcl-xl, Mcl-1, Bad, p-Bad, Bax and cytosolic cytochrome C (cytosolic) were observed in AGS cells treated with various concentrations of 13-AC. (**C**) Effects of three different mitochondrial permeability transition inhibitors, ArA, CyA and TFZ, on the cell viability upon treatment with 13-AC. The mock- and 13-AC-treated cells were combined with different inhibitors as indicated for 24 h, followed by examination of the cell viability by MTT assay. The data shown are representative of three independent experiments (^#^ *p* < 0.05). (**D**) Examination of the differentiated expression levels of mitochondrial-related apoptosis pathway proteins in response to the different inhibitors as indicated in 13-AC treated cells. β-actin was used as the protein loading control.

As the change in the mitochondrial membrane potential (ΔΨm) is well-defined as a role involving in initiation of mitochondrial-related apoptosis, we measured the change in ΔΨm induced by 13-AC using JC-1 dye. As a result, fluorescence microscopy showed significantly reduced red fluorescence signals (JC-1 aggregation) and increased green fluorescence signals (JC-1 monomer) in the 13-AC-treated AGS cells, suggesting a loss of ΔΨm upon 13-AC treatment (Figure 3A). Next, we explored the potential mechanism of 13-AC-induced apoptosis in AGS cells. To address this issue, several mitochondrial-related apoptotic proteins, including Bcl-2, Bcl-xl, Mcl-1, Bad, p-Bad, Bax, Bid and cytosolic cytochrome C, were investigated by western blotting using specific antibodies as indicated. The expression levels of Bax, Bad and cytosolic cytochrome C were significantly increased in a dose-dependent manner in the 13-AC-treated AGS cells. In contrast, the expression levels of Bcl-2, Bcl-xl, Mcl-1, and p-Bad were decreased upon 13-AC treatment (Figure 3B). It has been reported that gene members of the Bcl-2 family regulate the mitochondrial cell death pathway; in particular, the stoichiometries of Bax (pro-apoptotic gene) and Bcl-2 (anti-apoptotic gene) are important for cytochrome C release as well as the following downstream activation of caspase protein. In this study, increased expression levels of Bax and cytochrome C were detected in the 13-AC-treated cells, implying that the 13-AC-induced apoptosis was associated with activation of the mitochondrial-related pathway in AGS cells. Furthermore, three different mitochondrial permeability transition inhibitors (aristolochic acid; ArA, cyclosporine A; CyA and trifluoperazine; TFZ) were used for treatment prior to treatment with 13-AC in AGS cells. As expected, these inhibitors inhibited the 13-AC-induced cell apoptosis (Figure 3C). In addition, several mitochondrial-related apoptotic proteins, including cytosolic cytochrome C, Bax, Bcl-2 and Bcl-xl proteins, were detected by western blotting. The expression levels of Bcl-2 and Bcl-xl were increased in the 13-AC-treated cells when the cells were pretreated with CyA, ArA and TFZ. In contrast, the expression levels of Bax and cytosolic cytochrome C were decreased in the 13-AC-treated cells when the cells were pretreated with CyA, ArA and TFZ (Figure 3D). Taken together, these results indicated that mitochondria dysfunction-mediated apoptosis was involved in 13-AC-induced cell apoptosis.

### 2.4. 13-AC Activates the Caspase-Dependent Pathway Causing Cell Apoptosis in AGS Cells

**Figure 4:**
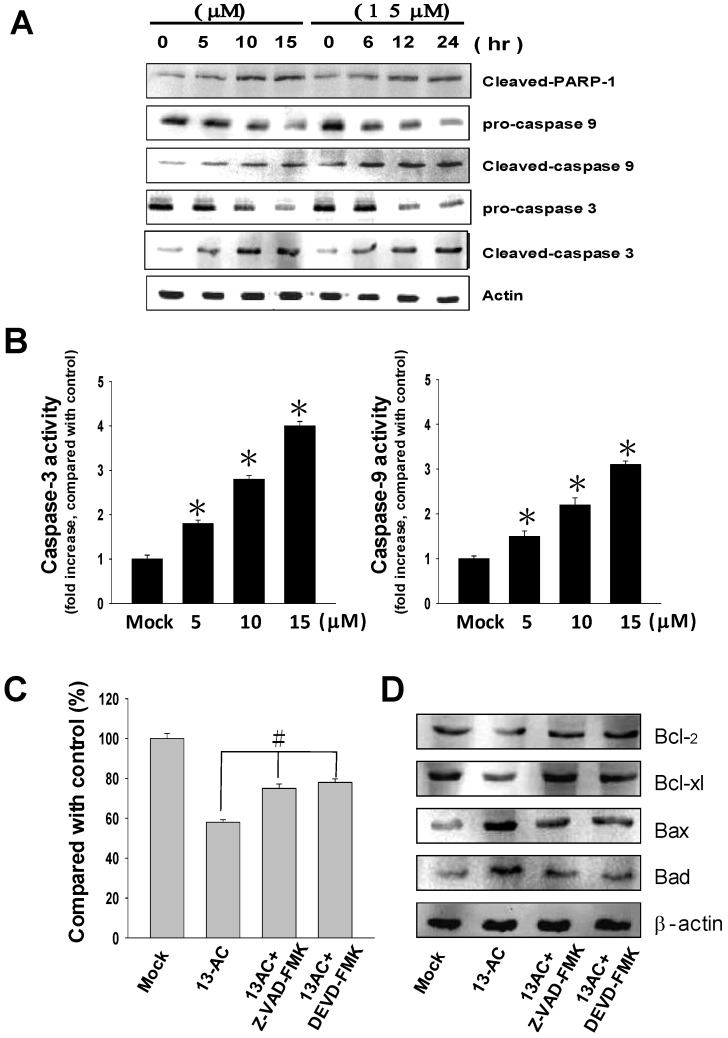
13-AC induced apoptosis through activation of the caspase cascade pathway. (**A**) AGS cells were treated with 13-AC either at various concentrations of 0, 5, 10, and 15 μM for 24 h or at the concentration of 15 μM for different time periods of 6, 12, and 24 h. The harvested cell lysates were then examined for PARP-1, caspase-3 and caspase-9 by western blotting using specific antibodies as indicated. β-actin was used as the protein loading control. (**B**) Caspase-3 and caspase-9 activities were increased after 13-AC treatment. The results shown are representative of three independent experiments (^#^ *p* < 0.05, * *p* < 0.001 as compared with the control). (**C**) Two caspase inhibitors, Z-VAD-FMK (caspase-3 inhibitor) and Z-DEVD-FMK (caspase-9 inhibitor), were added simultaneously with 13-AC to AGS cells. The cells were then harvested and subjected to MTT assay for the evaluation of cell viability. The related cell viabilities were determined from three independent experiments (^#^ *p* < 0.05 as compared with the control). (**D**) Examination of protein levels for some mitochondrial-related apoptotic proteins, including Bcl-2, Bcl-xl, Bax and Bad proteins. The mock- and 13-AC-treated cell lysates were harvested and subjected to western blot analyses using specific antibodies as indicated.

Caspase, another kinase protein that plays an important role in the regulation of cell apoptosis, was investigated in addition to three mitochondrial-related apoptosis proteins as described above [20,32]. It is believed that activation of both caspase-3 and caspase-9 stimulates mitochondrial cell death signals [33]. In this study, we further investigated the effects of 13-AC on the activation of caspase-3, caspase-9 and PARP cleavage by western blotting using specific antibodies as shown in the Materials and Methods section. In Figure 4A, the western blotting results show up-regulated expression levels of cleaved-PARP (89 kDa proteolytic fragments), cleaved-caspase-3 and cleaved-caspased-9 in 13-AC-treated cells. Down-regulated expression levels of pro-caspase-3 and pro-caspase-9 after 13-AC treatment were observed. In addition, 13-AC treatment increased the caspase-3 and caspase-9 activities (Figure 4B). These results showed that 13-AC could activate the caspase-dependent pathway.

To gain a comprehensive understanding of 13-AC-induced cell apoptosis through caspase activation, cells were pre-treated with either Z-LEHD-FMK (caspase-9 inhibitor) or Z-DEVD-FMK (caspase-3 inhibitor) prior to 13-AC treatment. The results showed that Z-LEHD-FMK and Z-DEVD-FMK significantly inhibited the 13-AC-induced cell apoptosis (Figure 4C). Taken together, these results suggested that both caspase-3 and caspase-9 proteins are involved in the 13-AC-induced cell apoptosis in AGS cells.

### 2.5. 13-AC Induces Activation of the p38 and JNK Pathways and Suppression of PI3K/AKT

**Figure 5:**
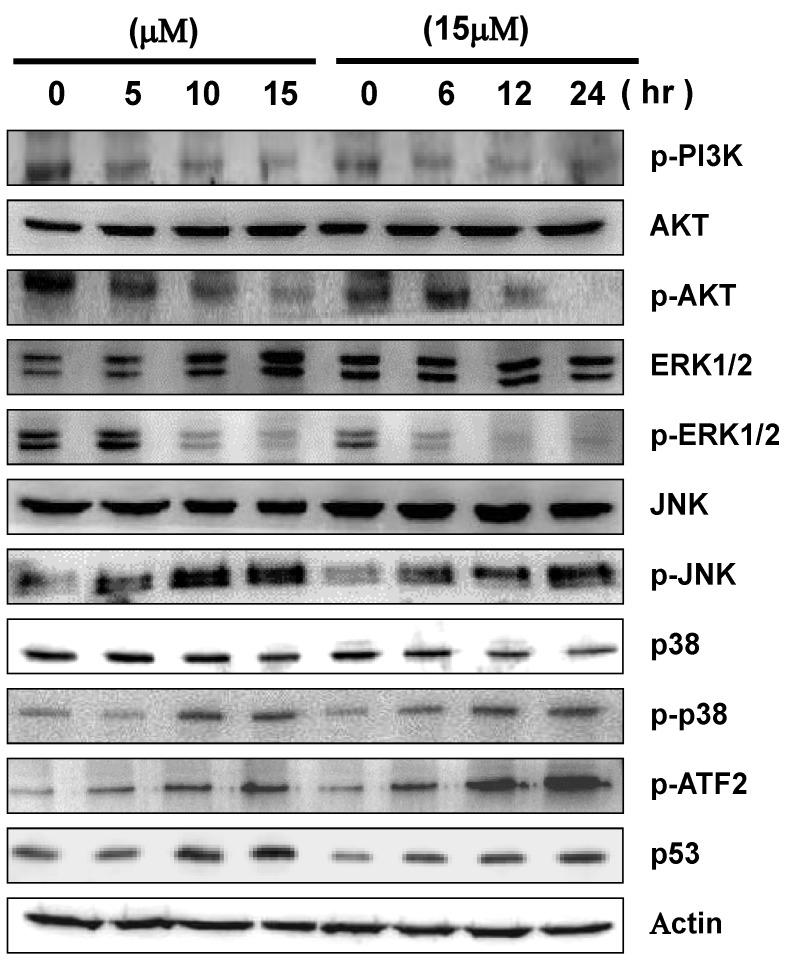
Effects of 13-AC on the cytotoxicity of AGS cells through MAPK pathway-related proteins. AGS cells were treated with 13-AC at various concentrations of 0, 5, 10, and 15 μM for 24 h or treated with 13-AC at the concentration of 15 μM for 6, 12, and 24 h. The cells were then harvested and subsequently analyzed for the differential expression levels of proteins by western blotting. β-actin was used as the protein loading control.

Mitogen-activated protein kinases (MAPKs) signal pathways have been demonstrated to play important roles in various biological processes, such as cell proliferation, differentiation and apoptosis [34,35]. Herein, we examined the expression levels of proteins involved in the MAPKs signal pathways in order to investigate the potential effects of 13-AC-induced apoptosis through activation of the MAPKs signaling pathways. As shown in Figure 5, the protein expressions of phosphorylated p38 and JNK were significantly activated in a dose- and time-dependent manner. In contrast, the expression levels of non-phosphorylated AKT, ERK, JNK and p38 were not changed upon 13-AC treatment, nor for treatment with 15 μM of 13-AC for various durations. Moreover, down-regulated expression levels of phosphorylated forms of PI3K, AKT and ERK1/2 proteins were also observed in 13-AC-treated cells in both dose- and time-dependent manners. In addition, the expression levels of p53 and p-ATF2 were also up-regulated upon 13-AC treatment.

**Figure 6:**
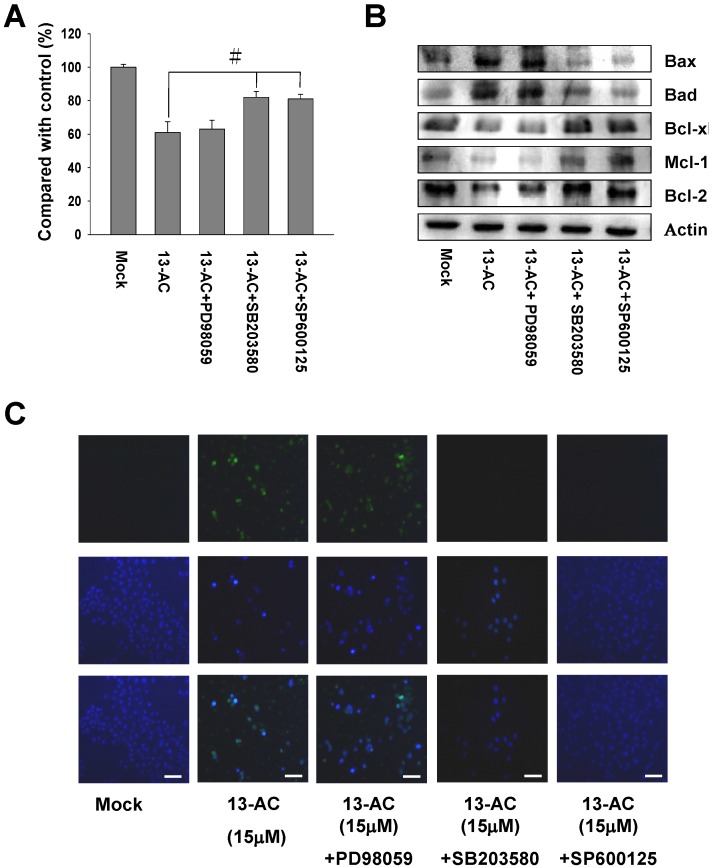
Inhibition of p38 and JNK activities rescued the cell cytotoxicity of AGS cells caused by 13-AC. (**A**) Two inhibitors, SB203580 (p38-specific inhibitor) and SP600125 (JNK-specific inhibitor), were added to AGS cells treated with 13-AC and the cell viability was detected by MTT assay as described above. An increased cell viability of the 13-AC-treated AGS cells was observed following addition of SB203580 and SP600125, but not PD98059. The results shown are representative of three independent experiments (^#^ *p* < 0.05 as compared with the control). (**B**) Validation of the expression levels of mitochondrial-related proteins affected by two inhibitors. β-actin was used as the protein loading control. (**C**) Decrease in the apoptotic AGS cells characterized by TUNEL and DAPI staining assays. TUNEL and DAPI staining showed decreases in the 13-AC-induced apoptotic characteristics when treated with SB203580 and SP600125. Scale bars = 50 μm.

### 2.6. Inhibition of p38 and JNK Activities Rescued the Cell Cytotoxicity of AGS Cells by 13-AC

To further explore whether 13-AC-induced apoptosis is mediated through the MAPKs pathways, three different inhibitors (PD98059, SB03580 and SP600125) were added to elucidate the MAPKs-activated cell apoptosis upon 13-AC treatment. As shown in Figure 6A, an increasing cell viability was observed (60%–80%) upon treatment with SB203580 as well as similar effects of SP600125 at the concentration of 15 μM in 13-AC-treated AGS cells. In contrast, the cell viability of 13-AC-treated AGS cells was not increased after pre-treatment with PD98059 (a MEK-specific inhibitor). As SB203580 is a p38-specific inhibitor and SP600125 is a JNK-specific inhibitor, we concluded that 13-AC induced cell apoptosis in AGS cells through the p38 and JNK signaling pathways. Furthermore, the expression levels of Bcl-2, Bcl-xl and Mcl-1 proteins were increased, while on the other hand, the expression levels of Bax and Bad proteins were decreased in 13-AC-treated cells when the cells were pretreated with SB203580 and SP600125 (Figure 6B). In addition, TUNEL and DAPI assays were performed to detect DNA fragmentation after 13-AC-induced apoptosis. Positively TUNEL- and DAPI-stained AGS cells after 13-AC treatment are shown in Figure 6C. Upon pre-treatment with SB203580 or SP600125, the amount of positively TUNEL- and DAPI-stained AGS cells was significantly decreased in comparison with the negative control, which was 13-AC-treated AGS cells (Figure 6C). Altogether, these results indicated that the p38 and JNK pathways are partially involved in the 13-AC-induced apoptosis of AGS cells.

## 3. Discussion

The current study demonstrated that the anti-cancer effect of 13-AC is related to its cell cytotoxic effects, and apoptosis is induced in AGS cells. 13-AC treatment clearly reduced the cell viability of AGS cells in a dose-dependent manner, as shown by MTT assay. Besides, colony formation assays and wound-healing assays were employed for identification of the cytotoxic effects of 13-AC on AGS cells (Figure 1). The apoptotic morphological characteristics of AGS cells induced by 13-AC were also observed by TUNEL/DAPI staining and Annexin V/PI staining assays using flow cytometry, which showed that both early and late apoptosis occurred upon 13-AC treatment (Figure 2).

### 3.1. 13-AC Induces Apoptosis and Causes Mitochondria Dysfunction in AGS Cells

There is growing interest in cell biology in terms of evaluating changes in the mitochondrial membrane potential so as to define the role in initiating apoptosis and the cell cycle. In this study, the mitochondrial membrane potential (ΔΨm) was assessed using JC-1 dye. 13-AC-treated AGS cells showed a significant reduction in red fluorescence and increased signals in green fluorescence, suggesting a loss of mitochondrial membrane potential due to treatment with 13-AC (Figure 4A).

Bcl-2 family members regulate the mitochondrial cell death pathway [36]; in particular, the stoichiometries of Bax (pro-apoptotic member) and Bcl-2 (anti-apoptotic member) are critical for cytochrome C release and the following downstream caspase activation [37,38,39]. In this study, we comprehensively investigated the differentiated expression levels of several mitochondrial-related apoptosis proteins, including Bcl-2, Bcl-xl, Mcl-1, Bad, p-Bad, Bax and cytosolic cytochrome C, to explore the potential mechanisms of 13-AC-induced apoptosis through mitochondria-dependent pathways. The results are shown in Figure 4B. The expression levels of Bax, Bad and cytosolic cytochrome C were increased in time- and dose-dependent manners following 13-AC treatment. In contrast, the expression levels of Bcl-2, Bcl-xl, Mcl-1 and p-Bad were decreased upon 13-AC treatment. These results showed that 13-AC treatment induced cell apoptosis through activation of pro-apoptotic Bax and Bad and suppression of anti-apoptotic proteins such as Bcl-2 and Bcl-xl, which have been reported to regulate cytochrome C release from mitochondria. 

Here, we demonstrated that the mitochondrial membrane potential in AGS cells was attenuated upon 13-AC treatment (Figure 5). These results are rationally linked with the western blotting data of these mitochondrial apoptotic events, such as inhibition of Bcl-2, Bcl-xl, Mcl-1, and p-Bad expressions and simultaneous enhancement of Bax and Bad expressions. Subsequently, Bax inserts into the mitochondria outer membrane and releases cytochrome C from mitochondrial inter-membrane spaces. Then, caspase-9 is activated, further activating the downstream effector caspase-3 in order to cleave poly (ADP-ribose) polymerase-1 (PARP-1), which is further activated.

Activation of both caspase-3 and caspase-9 are believed as toward mitochondrial apoptosis signals [40]. It is also well-known that the caspases are proteolytic activation during apoptosis. Upon receiving proapoptotic signals, the activation of caspase-3 requires the activation of initiator caspases, such as caspase-8 or caspase-9 [41]. In this study, we investigated the changes in the expression levels of caspase-3 and caspase-9 upon 13-AC treatment in AGS cells. The western blotting data showed that both cleaved-caspase-3 and cleaved-caspase-9 were up-regulated in 13-AC-treated cells (Figure 5). Similarly, the cleaved form of PARP-1 (with a molecular size of 89 kDa) was elevated upon 13-AC treatment. This observation is consistent with a report showing that PARP-1 is cleaved by caspase during apoptosis [33]. Furthermore, two caspase inhibitors, Z-VAD-FMK and Z-DEVD-FMK, were added simultaneously with treatment with 13-AC in AGS cells. As per the results shown, both caspase inhibitors elevated the cell viability and inhibited the 13-AC-induced cell cytotoxicity (Figure 5B). Taken together, these results revealed that the mitochondrial-dependent apoptosis pathway and activation of caspase are involved in 13-AC-induced cell apoptosis. Interestingly, these findings are similar to the possible mechanisms of apoptosis induction by another compound extracted from soft coral, 11-dehydrosinulariolide, in oral carcinoma cells and sinularin in melanoma cells, as described in our previous studies [18,42].

### 3.2. The MAPK Signaling Pathways are Involved in 13-AC-Induced Apoptosis

It is well-known that mitogen-activated protein kinases (MAPKs) signaling pathways regulate cell proliferation, differentiation and apoptotic cell death in response to a variety of stresses or stimuli [43,44]. The three well-characterized subfamilies of MAPKs are extracellular signal regulated kinase 1 and 2 (ERK1/2), c-Jun NH2-terminal kinase (JNK) and p38 [45]. In this study, phosphorylated p38 and JNK were significantly activated, but phosphorylatedERK1/2 was down-regulated upon 13-AC treatment. The expression levels of non-phosphorylated ERK1/2, p38 and JNK did not change in comparison with DMSO-treated cells (Figure 6).

The p38 and JNK pathways are activated in response to various chemicals and stresses [46]. Initiation of the p38 and JNK pathways is required for induction of cellular apoptotic pathways, as shown in several studies [47,48]. Chemical stress-induced p38 and JNK activation was shown to regulate the cell cycle and cell apoptosis through modulation of the p53 tumor suppressor protein [49,50,51]. The p53 protein was decreased pro-survival Bcl-2 proteins and elevated pro-apoptosis Bax protein expression [52,53]. The ATF2 protein was phosphorylated either by p38 or JNK [54]. As shown in Figure 6, the expression levels of p53 and phosphorylated ATF2 were increased upon 13-AC treatment. Therefore, our results suggested that p38 and JNK signaling, at least partially, is responsible for 13-AC-induced apoptosis of AGS cells.

It is well-known that ERK1/2 plays a crucial role in mediating different cell functions, such as cell survival, cell cycle progression, apoptosis and autophagy. Phosphorylated ERK1/2 resulted in nuclear translocation and activation of substrates, promoting cell proliferation and inhibiting proapoptotic signals [55]. Many studies have shown that ERK1/2 has effects of the proliferation and protection of cells exposed to oxidative stress [56]. In this study, 13-AC significantly reduced phosphorylated ERK1/2 in AGS cells. Additionally, inhibition of ERK1/2 activation downregulated the expressions of Bcl-2 and Bcl-xl [57], and ERK1/2/Bcl-2 signaling is believed to be a potential therapeutic target for cancer cells [58]. 13-AC-mediated gastric carcinoma cell apoptosis was closely associated with down-regulation of the ERK1/2 signaling pathway.

The phosphatidylinositol 3-kinase/protein kinase-B (PI3K/AKT) cell signaling cascade is one of the most important intracellular pathways and is frequently activated in diverse cancers, regulating cell proliferation, cell differentiation, cellular apoptosis and cancer cell survival [59]. AKT is an important part of PI3K signaling. The activation of AKT is caused by PI3K- and PDK1-mediated phosphorylation in the catalytic domain at threonine 308. AKT regulates downstream targets in the PI3K pathway, such as TSC2, and outside of the PI3K pathway, such as Bcl-2-associated proteins and glycogen synthase kinase-3B [60].

Many studies have indicated that downregulation of the PI3K/AKT signaling pathway induces cell apoptosis [61,62]. The expression levels of phosphorylated PI3K and AKT were decreased after 13-AC treatment. Therefore, our results suggested, at least partially, that 13-AC induced apoptosis of AGS cells through downregulation of PI3K/AKT signaling.

## 4. Materials and Methods

### 4.1. Materials

13-AC was extracted from cultured Formosa soft coral *Sarcophytoncrassocaule* following the protocol described elsewhere [63] and dissolved in DMSO. A mitochondria/cytosol fractionation kit was obtained from BioSource International (Camarillo, CA, USA). Chemiluminescent HRP substrate was purchased from Pierce (Rockford, IL, USA). An annexin V–FITC Apoptosis Detection kit was obtained from Pharmingen (San Diego, CA, USA). A JC-1 (5,5,6,6-tetrachloro-1,1,3,3-tetraethylbenzimidazolcarbocyanine iodide) fluorescent kit was obtained from Biotium (Hayward, CA, USA). A DeadEnd™ Fluorometric TUNEL fluorescent kit and a 4′-6-diamidino-2-phenylindole (DAPI) fluorescent kit were obtained from Promega (Madison, WI, USA). 3-(4,5-Dimethylthiazol-2-yl)-2, 5-diphenyltetrazolium bromide (MTT), dimethyl sulfoxide (DMSO), cyclosporine A (CyA), aristolochic acid (ArA), trifluoperazine (TFZ), Z-DEVD-FMK (caspase-3 inhibitor), Z-LEHD-FMK (caspase-9 inhibitor), protease inhibitor cocktail and rabbit anti-human β-actin antibodies were obtained from Sigma (St Louis, MO, USA). Goat anti-rabbit and horseradish peroxidase conjugated IgG and PVDF (polyvinylidenedifluoride) membranes were obtained from Millipore (Bellerica, MA, USA).

### 4.2. Cell Culture and 13-AC Treatment

AGS gastric carcinoma cells were obtained from the Bioresource Collection and Research Center (CBC, Food Industry Research and Development Institute, Hsinchu, Taiwan). Cells were cultured as described in our previous study [64]. AGS cells were maintained in F-12K media supplemented with 10% FBS, 100 U/mL penicillin and 100 μg/mL streptomycin in a humidified incubator with 5% CO_2_ and 95% air at 37 °C. Dimethylsulfoxide (DMSO) was used to dissolve 13-AC. AGS cells were treated with different concentrations of 13-AC (0, 2.5, 5, 10, 15 and 20 μM) and harvested after incubation for 24 h. All the experiments were repeated three times.

### 4.3. MTT Assay for Cellular Cytotoxicity

The cytotoxic effect of 13-AC on AGS cells was measured by MTT proliferation assay according to a previous report [19]. AGS cells were seeded at 1 × 10^4^ per well in 96-well plates before treatment with various concentrations of 13-AC. The cells were treated with various concentrations of 13-AC (0, 2.5, 5, 10, 15 and 20 μM) in 200 μL of serum complete media. After 24 h of treatment, MTT solution (1 mg/mL in PBS) was added to each well. The plates were then incubated at 37 °C for 4 h, and reduced purple-blue MTT formazan crystals were solubilized by adding 200 μL of DMSO to each well. The absorbance was measured at 595 nm using a microplate ELISA reader, with DMSO used as the blank.

### 4.4. Wound-Healing Assay

The anti-migratory effects of 13-AC on AGS cells were examined by a wound-healing assay according to a previous report [42,65]. AGS cells were seeded in 6-well plates. After the AGS cells grew to confluence, an artificial scratch was made with a tip in each of the wells. Cells were washed with PBS and refreshed with FBS-containing medium. Images of the experimental groups (0, 5, 10 and 15 μM 13-AC) were acquired at 0, 6, 12 and 24 h after treatment with 13-AC.

### 4.5. Colony Formation Assay

The colony formation assay was performed according to a previous report [66]. AGS cells were seeded in 24-well plates at a density of 2000 cells/well. After incubation for 24 h, cells were treated with various concentrations (0, 5, 10, 15 and 20 μM) of 13-AC in 2 ml of serum complete media. After incubation for 10 days, the colonies were washed with PBS and fixed with methanol for 15 min and stained with 0.15% crystal violet. The colonies were counted and scanned with a high-resolution scanner Scan Maker 9800XL (MiCROTEK, Hsinchu, Taiwain).

### 4.6. Antibody and Western Blot Analyses

Rabbit anti-human Bax, ERK, JNK and p-JNK antibodies were obtained from ProteinTech Group (Chicago, IL, USA). Rabbit anti-human antibodies against p-PI3K, AKT, p-AKT, PARP, pro-caspase-3, cleaved-caspase-3, pro-caspase-9, cleaved-caspase-9, Bcl-xl, Bcl-2, Mcl-1, Bad, p38, p-p38, p-ERK, p-ATF2 and p53 were obtained from Cell Signaling Technology (Danvers, MA, USA). Rabbit anti-human antibodies against p-Bad and cytochrome C were obtained from Epitomics (Burlingame, CA, USA). Rabbit anti-human β-actin antibodies were obtained from Sigma (St Louis, MO, USA). Goat anti-rabbit and horseradish peroxidase conjugated IgG was obtained from Millipore (Bellerica, MA, USA). Immunoblotting was performed as previously described [19]. The treated samples and the control samples (25 μg) were separated by 12.5% SDS-polyacrylamide gel electrophoresis (SDS-PAGE). The proteins on the gel were then transferred to PVDF membranes. The PVDF membranes were incubated with the primary antibody (1:1000 dilutions in 2% dehydrated skim milk) at 4 °C overnight. Then, incubation at 4 °C was performed for 2 h with the secondary antibodies (goat anti-rabbit or goat anti-mouse and horseradish peroxidase conjugate, 1:5000 dilution in 2% dehydrated skim milk). The blots were detected through chemiluminescence using enhanced ECL western blotting kit.

### 4.7. Annexin V-FITC Apoptosis Assay

Cell apoptosis was measured by Annexin/PI double staining as previously reported [67]. A total of 1 × 10^6^ cells were seeded onto 5-cm Petri dishes and treated with 0.1%DMSO as the vehicle or 10 and 15 μM of 13-AC for 24 h. The cells were subsequently collected and fixed in 70% cold ethanol at 4 °C overnight. The cells were then stained with 10 μg/mL Annexin V-FITC and 5 μg/mL propidium iodide (PI) for 30 min at 37 °C. A FACSCalibur flow cytometer and Cell-Quest software (Becton-Dickinson, Mansfield, MA, USA) were used to analyze the apoptotic cells.

### 4.8. Immunofluorescence Microscopy

AGS cells (1 × 10^5^ cells/well) cultured in 12-well plates were pretreated with 0, 10 and 15 μM 13-AC for 24 h. Then, the Annexin V-FITC Apoptosis staining reagent was added to the cells according to manufacturer’s protocol and incubated for 15 min, then observed under fluorescence microscopy (Olympus IX71 CTS, Chinetek Scientific, Hong Kong, China). The DeadEnd™ Fluorometric TUNEL System (Promega, Madison, WI, USA) was used to detect nuclear DNA fragmentation according to the manufacturer’s manual. The fluorescein-12-dUTP-labeled DNA can then be visualized directly under fluorescence microscopy (Olympus IX71 CTS, Chinetekcientific, Hong Kong, China). The cells were photographed. A fluorescence microscope was also used to identify the condense nuclei and DNA chromatin fragmented that were stained with DAPI [29].

### 4.9. Mitochondrial Membrane Potential (ΔΨm) Assay Using Fluorescence Microscopy

Changes in the mitochondrial membrane potential (ΔΨm) after treatment with different concentrations of 13-AC were examined by staining with JC-1 (5,5,6,6-tetrachloro-1,1,3,3-tetraethylbenzimidazolcarbocyanine iodide) dye (Biotium, Hayward, CA, USA). AGS cells were pretreated with 13-AC and incubated with 10 mg/mL JC-1 at 37 °C for 30 min in the dark. Cells were washed twice with PBS and observed under fluorescence microscopy (Olympus IX71 CTS, Chinetek Scientific, Hong Kong, China).

### 4.10. Inhibitors Assessment

To further determine the effects of p38, ERK, and JNK on 13-AC-induced cell proliferation arrest, a total of 1 × 10^5^ cells seeded in a 24-well plate were pre-incubated for 2 h with specific inhibitors for p38 (SB239063), ERK (PD98059), and JNK (SP600125) prior to 13-AC administration. Afterwards, the cell viability rate was determined by MTT assay.

### 4.11. Statistical Analysis

The results of the MTT assay, colony formation assay, cell cycle distribution analysis, caspase-3 activity and caspase-9 activity were analyzed by Student’s test. *p* < 0.05 was considered statistically significant.

## 5. Conclusion

In conclusion, our study results suggest that 13-AC induces apoptosis through the activation of p38 and JNK and suppression of PI3K/AKT so as to affect the expression levels of mitochondrial apoptotic proteins. Therefore, the results may provide potential pharmacological evidence that 13-AC possesses anti-gastric carcinoma effects, and may be employed as a potential chemotherapeutic agent.

## Supplementary Files

Supplementary File 1
