# Supplementary material for: 13-Acetoxysarcocrassolide Induces Apoptosis on Human Gastric Carcinoma Cells Through Mitochondria-Related Apoptotic Pathways: p38/JNK Activation and PI3K/AKT Suppression
Source: Mar Drugs. 2014 Oct 23;12(10):5295–315. doi: 10.3390/md12105295 (PMC4210900; doi:10.3390/md12105295)
Supplement: Supplementary File 1 [file marinedrugs-12-05295-s001.pdf]

Supplementary Information

Figure S1. The cell viability of two gastric cancer cell lines, SNU-1 and NCI-N87 cells, were suppressed in a dose-dependently manner upon treatment with 13-AC. The SNU-1 and NCI-N87 cells were treated without or with the13-AC at the final concentration between 2.5 μM and 20 μM for 24h. The cells were then harvested for the MTT assay as described in the Materials and Methods. Data shown here is representative of three independent experimental results.

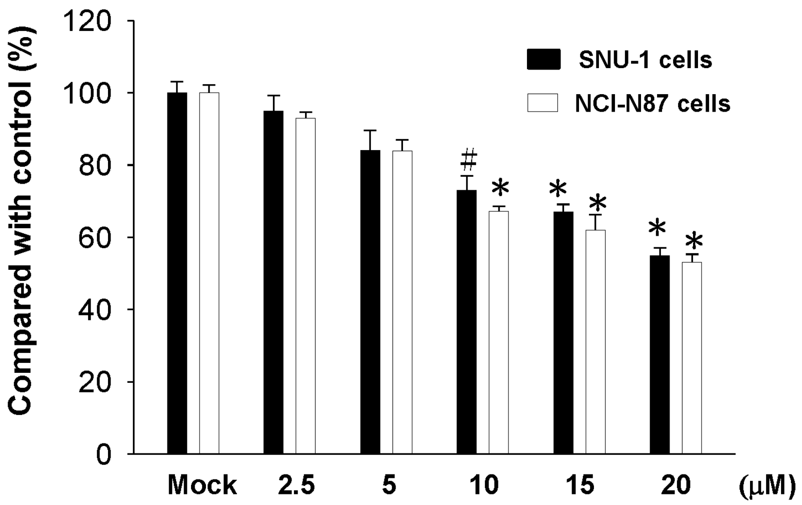

© 2014 by the authors; licensee MDPI, Basel, Switzerland. This article is an open access article distributed under the terms and conditions of the Creative Commons Attribution license (http://creativecommons.org/licenses/by/3.0/).
